# Pre-ischemic exercise prevents inflammation and apoptosis by inhibiting MAPK pathway in ischemic stroke

**DOI:** 10.1515/tnsci-2022-0268

**Published:** 2022-12-29

**Authors:** Zhen-Kun Gao, Xin-Ya Shen, Yu Han, Yi-Sha Guo, Kai Li, Xia Bi

**Affiliations:** Department of Graduate School, Shanghai University of Traditional Chinese Medicine, Shanghai 201203, China; Department of Rehabilitation Medicine, Shanghai University of Medicine & Health Sciences Affiliated Zhoupu Hospital, No. 1500 Zhouyuan Road, Pudong New District, Shanghai 201318, China

**Keywords:** MCAO, pre-ischemic exercise, MAPK, inflammation, apoptosis

## Abstract

**Introduction:**

Mitogen-activated protein kinase (MAPK) pathway is a major mechanism of acute brain damage in ischemic stroke. Pre-ischemic exercise is an effective method to reduce ischemic injury. However, the regulation by pre-ischemic exercise of MAPK pathway and associated mechanisms in animal models remains unclear.

**Materials and methods:**

In this study, Male SD rats were randomly divided into sham group, middle cerebral artery occlusion (MCAO) group, and exercise plus MCAO (EX + MCAO) group for 21 days, and then was established by MCAO. Longa score was used to measure neurological deficits at 0, 1, 2, and 3 days after MCAO. Hematoxylin and eosin staining was used to observe the brain injury. The expression of MAPK pathway was quantified by western blot. The M1 microglia protein was quantified by western blot and immunofluorescence, and the level of inflammatory factor was measured by enzyme-linked immunosorbent assay. TUNEL staining and western blot were used to measure apoptosis.

**Results:**

In the current study, we observed that pre-ischemic exercise effectively decreased infarct volume, neurological deficit score and brain injury in MCAO rats through suppressing the activation of p-JNK and p-ERK1/2. Further investigation revealed that pre-ischemic exercise decreased M1 microglia activation and the serum level of TNF-α and IL-1β. In addition, the increased number of TUNEL-positive cells and Bax/Bcl-2 ratio also were reversed by pre-ischemic exercise.

**Conclusions:**

Pre-ischemic exercise can alleviate inflammatory response and apoptosis by inhibiting the MAPK pathway in MCAO rats.

## 1 Introduction

Ischemic stroke is caused by vascular obstruction of the brain supply, characterized by high incidence rate, high disability rate, high recurrence rate, and high mortality rate [1]. There are currently no available treatment options for brain damage. Tissue plasminogen activator is the only FDA-approved agent, but its clinical application is limited due to its narrow therapeutic window and poor treatment outcomes [2]. Acute injury within 3 days of ischemia–reperfusion (I/R) injury is an important prognostic determinant. Among survivors, more than half required long-term rehabilitation due to physical disability and neurocognitive impairment [3].

Reducing acute brain injury after I/R injury and subsequent injury is critical, and exercise pre-conditioning is the most common treatment to address this problem. This protection through pre-ischemic exercise may include reducing cerebral edema and cerebral infarct volume [4], reducing brain neuroinflammation [5], and suppression of neuronal apoptosis [6]. In addition, regular exercise training is a promising public health strategy, which is easy to provide for patients and does not require high costs. A prospective cohort study found that pre-stroke physical activity (such as housework and heavy physical labor) at least three times a week was associated with better functional outcomes [7]. Although the mechanisms of pre-conditioning methods have been extensively tested in ischemic stroke, the underlying neuroprotective molecular mechanism of pre-ischemic exercise in early brain injury is still unclear.

Mitogen-activated protein kinase (MAPK) signaling pathway is a conserved pathway regulated by a series of protein kinases and MAPK phosphatases, which regulate the body’s response to external stimuli (such as cytokines secretion and environmental changes) [8]. MAPK pathway has three members, extracellular signal-regulated kinase (ERK), c-Jun N-terminal kinase (JNK), and p38 MAPK. ERK1/2 mediates metabolism, inflammation, and apoptosis. JNK and P38 MAPK are involved in cell apoptosis after I/R injury [9]. The activation of MAPK pathway alters the energy balance of the brain, followed by a series of pathological events, such as inflammation, excitotoxicity, apoptosis, and oxidative stress [10]. Inflammation is one of most important pathological mechanisms of acute I/R injury. As the major glial cell, microglia are responsible for immune regulation in ischemic stroke [11]. Microglia rapidly activate into M1 type and release inflammatory factors to cause cell death after brain injury. In addition, the activation of M1 microglia may lead to the initiation of apoptosis. Bax and Bcl-2 participate in necrotic cell death. Bax is one of the key proapoptotic genes of apoptosis, and Bcl-2 is one of the key executors of antiapoptotic.

Current findings have demonstrated that activated MAPK signaling pathway contributes to the activation of microglia and the release of proinflammatory factors in brain tissue after ischemic stroke. Among them, ERK1/2 appears to be primarily involved in cell apoptosis [12]. And pre-ischemic exercise can inhibit the expression of p-ERK1/2 [13]. Based on the aforementioned findings, we hypothesized that pre-ischemic exercise may play a significant role in regulating the response ischemic stroke by manipulating MAPK signaling pathway. Therefore, we conducted experiment to investigate the effects of exercise preconditioning (EP) on MAPK signaling pathway.

## 2 Materials and methods

### 2.1 Animals and experimental groups

Adult male Sprague-Dawley rats (6–8 weeks old, 250 ± 20 g) were (obtained from Shanghai SLRC Laboratory Animal Co., Ltd., Shanghai, China) housed under 22–24°C, 45–60% humidity, and 12:12 h light/dark cycle, with free access to food and water. After 3 days of adaptation, rats were randomly divided into three groups: the sham group, the middle cerebral artery occlusion (MCAO) group, and the EX + MCAO group (16 rats in each group). We used MCAO to induce cerebral ischemia. The sham group (served as controls) was subjected to the same surgical procedure except for MCAO.


**Ethics approval and consent to participate**: The research related to animals’ use has been complied with all the relevant national regulations and institutional policies for the care and use of animals. The experimental protocol of this study was approved by the Animal Ethical Committee of Shanghai University of Traditional Chinese Medicine.

### 2.2 Pre-exercise training protocol

Before the training, the EX + MCAO group rats underwent adaptive running training on an electric treadmill machine (BW-TDW709 Type-6-Lane Treadmill; Shanghai Biowill Co., Ltd, China) at a speed of 10 m/min for 3 days for 30 min a day. After the end of the adaptive exercise period, rats received a 21-day treadmill training at a speed of 20 m/min for 30 min a day for 6 days a week. The sham group and the MCAO group do not receive treadmill training, but let them run freely in the cage for 21 days.

### 2.3 Transient focal cerebral ischemia model

Briefly, rats were anesthetized with 1.5% isoflurane and the rat’s body temperature was maintained at 37°C. Rats were positioned supine and shaved at the median incision of the neck, and the right common carotid artery, external carotid artery, and internal carotid artery were isolated, followed by ligation of the distal external carotid artery and proximal common carotid artery. The 4–0 surgical filament (Rayward Life Technology Co., Ltd., Shenzhen, China) with silicone tip was advanced from external carotid artery into internal carotid artery lumen. After 90 min, the filament was withdrawn to establish reperfusion. During surgery, we used laser speckle imaging (Moor Instruments, Devon, USA) to detect the cerebral blood flow (CBF) of ischemic side. The rats detected with CBF will not be included in the subsequent detection. Other surgical procedures were the same except for MCAO in the sham group. After the rats recovered from anesthesia, they were scored based on a Longa score, and rats with a score of 1–3 points were successful models and were included in the study.

### 2.4 Neurological deficit assessment

Two researchers who were blinded to the test group performed neurobehavioral function tests on day 0, day 1, day 2, and day 3 after MCAO. The Longa score was used to evaluate neurological deficit: 0, no neurological symptoms; 1, unable to completely extend the front jaw on the contralateral side; 2, rotating while crawling and falling to the contralateral side; 3, unable to walk without assistance; and 4, unconsciousness.

### 2.5 Quantification of infarct volume

The rats were sacrificed 3 days after reperfusion. The fresh brain was stored at 20°C for 15 min and then cut into 2 mm thick coronal slices from the middle of the anterior pole and the optic chiasm along the tail. All sections were immediately placed into 0.1% 2,3,5-triphenyltetrazolium chloride (T8877, Sigma) solution, boiled in 37℃ water bath for 20 min, and took out for pictures. The total infarct volume was measured as the sum of the infarct size in each of the five sections.

### 2.6 Hematoxylin and eosin (HE) staining

The brain tissue was dehydrated, embedded in paraffin, and cut into 5 μm thick sections. The section was moved to a glass slide and dried and deparaffinized. HE staining was carried out according to standard protocols. Results were examined under a light microscope (Vectra 3, PerkinElmer, USA).

### 2.7 Western blot

Collected infarcted cortex tissue of rats was sonicated in the homogenization buffer (RIPA with protease cocktail inhibitor, phosphatase inhibitor, and phenylmethanesulfonyl fluoride). Protein concentration was determined using the bicinchoninic acid assay. Equal amounts of the samples and sample buffer were mixed and boiled for 10 min at 95°C and were subjected to sodium dodecyl sulfate polyacrylamide gel electrophoresis on 10% gel, and the proteins were transferred to a PVDF membrane (Millipore, PA, USA). The membrane was then incubated with the following primary antibodies overnight at 4℃: p38 (1:1,000, Abcam, ab170099), p-p38 (1:1,000, Abcam, ab195049), JNK (1:1,000, Abcam, ab179461), p-JNK (1:1,000, Abcam, ab124956), ERK1/2 (1:1,000, Abcam, ab184699), p-ERK1/2 (1:1,000, CST, 4370T), Iba-1 (1:1,000, Abcam, ab178847), iNOS (1: 1,000; Abcam, ab178945), SOD1 (1:1,000; Abcam, ab51254), Bax (1:500, Proteintech, 50599-2-Ig), and Bcl-2 (1:500, Proteintech, 12789-1-AP). After washing with TBST three times, the membrane was incubated with horseradish peroxide-conjugated secondary antibodies for 60 min at room temperature. The chemiluminescence signals were captured using EPSON Imaging System (EPSON; V300, Japan). Densitometric analysis was performed using Alpha Software (Alpha Innotech; alphaEaseFC, USA). β-Actin was used as a loading control.

### 2.8 Immunofluorescence staining

Frozen sections of tissue were fixed in 4% paraformaldehyde (PFA) overnight. The cortical tissue was fixed on the microtome, cut section (8 µm thick) in a cryostat (Leica CM1950), then collect every 3 microtome and store at 80°C. The sections were fixed with 4% PFA for 10 min at 26°C and blocked with 10% bovine serum albumin for 60 min. The slides were incubated with primary antibodies of Iba-1 (1:1,000, Abcam, ab178847) at 4°C overnight. After rinsing three times with phosphate buffered saline, slices were incubated with fluorescently coupled secondary antibody (anti-mouse, 1:3,000) for 60 min. Confocal microscope (LeicaTCS SP2) was used to photograph four fields of view (400×) of each area, and Image-Pro Plus 6.0 (Media Cyber Netics Inc., MD, USA) was used to evaluate the proportion of positive area.

### 2.9 Enzyme-linked immunosorbent assay (ELISA)

Serum was taken from blood samples at 72 h after MCAO. ELISA kits were performed to measure TNF-α and IL-1β (cat. no., RLB00, R&D Systems Inc., Minneapolis, MN, USA) levels in the serum by measuring the optical density at 450 nm. This experiment was repeated three times to calculate the average value.

### 2.10 TUNEL staining

TUNEL assay was carried out with the detection kit (Roche, Switzerland) to incubate the sample and TUNEL reaction solution at 37°C for 1 h and counterstained with 4′,6-diamidino-2-phenylindole (Beyotime, C1002, China) for 5 min. A fluorescence microscope (Vectra 3) was used to observe and photograph the brain sections. All counting procedures were calculated by an investigator blinded to experiment design.

### 2.11 Statistical analysis

**Figure 1:**
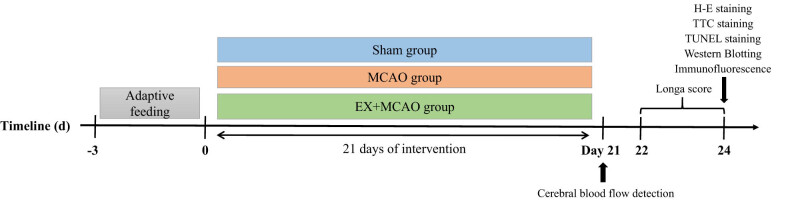
Experimental design. Before dividing into different groups, rats were required to be reared adaptively for 3 days. Each group had undergone intervention during 21 days. Neurological test started on day 0, day 1, day 2, and day 3 after sham or MCAO operation. The biochemical test was performed after the rats were sacrificed 3 days after MCAO.

Data were expressed as mean ± standard deviation using GraphPad Prism 8.0 software. IBM SPSS statistics 25 software was used in the study. Data were analyzed by one‐way analysis of variance followed by least significant difference multiple comparison tests as a post hoc comparison, Mann–Whitney *U* test or Kruskal–Wallis *H* test was performed for non-parametric analysis. Data were considered statistically significant when *P* < 0.05 (Figure 1).

## 3 Results

### 3.1 Successfully established cerebral I/R model

**Figure 2:**
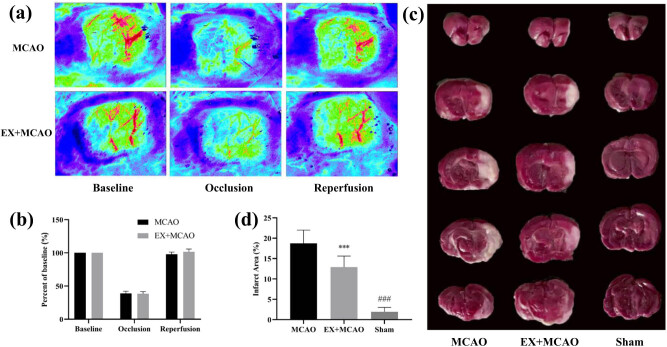
Successful establishment of MCAO model. (a) Laser speckle imaging to measure the average CBF as determined by laser Doppler analysis at pre-stroke, occlusion, and reperfusion CBF from the MCAO group and the EX + MCAO group. (b) Quantification of the CBF. *n* = 4/group. Data were expressed as the mean ± SD. (c) Representative images of TTC staining. The cerebral infarction areas are white. (d) Quantification of infarct volume in each group. *n* = 4/group, ***P < 0.001 vs sham group. Data were expressed as mean ± SD.

To ensure the same injury in rat models, we monitored the CBF of experimental rats by laser Doppler flowmetry before modeling and inserting filament and after removing inserting filament. The color of the right brain map changes to blue or cyan (Figure 2a), and the CBF quantitative analysis shows that a 60% reduction is a sign of successful operation (Figure 2b). After reperfusion, the CBF of two groups almost returned to normal level. The volume of infarct in each group was measured after staining with 2,3,5-triphenyltetrazolium chloride (Figure 2c and d). The infarct volume of MCAO rats was increased compared to sham rats. MCAO rats treated with pre-ischemic exercise showed the opposite outcomes.

### 3.2 Pre-ischemic exercise reduced neurological scores and brain injury

In MCAO models, the Longa scoring system was often used to evaluate the neurological impairment.

**Figure 3:**
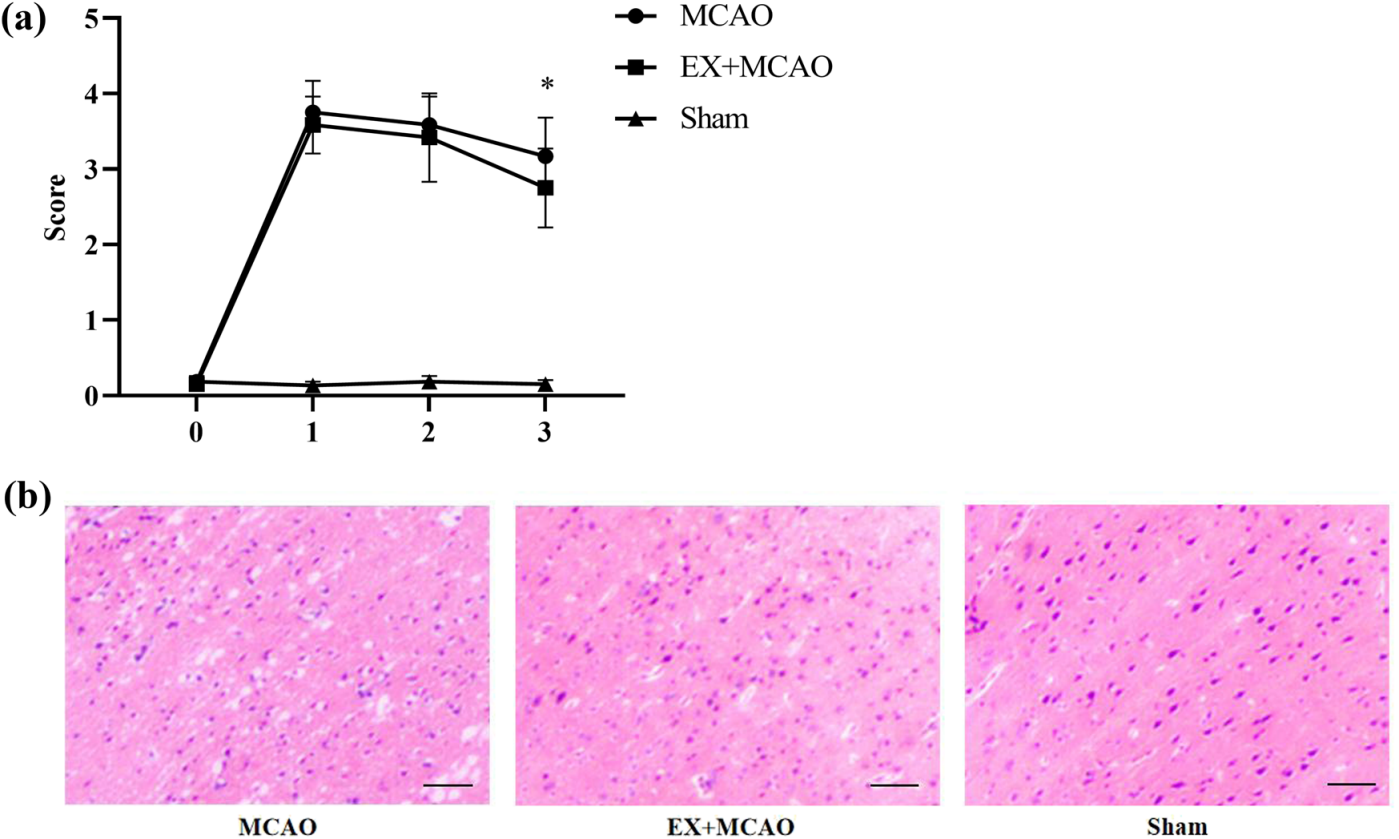
Effects of pre-ischemic exercise on neurological impairment and brain injury in MCAO rats. (a) Longa scores were assessed on 0, 1, 2, and 3 days after MCAO operation. *n* = 6/group, *P < 0.05 vs MCAO group. Data were expressed as mean ± SD. (b) Representative images of HE staining. Histopathological changes including structural disorders and neuronal damage. *n* = 6/group, scale bar represents 500 μm.

The rats in each group underwent behavioral test at 0, 1, 2, and 3 days after MCAO (Figure 3a). Rat models of MCAO exhibited increased neurological function scores compared with sham rat (*P* < 0.05). This observation was eliminated in the rat treated with pre-ischemic exercise on the third day of MCAO (*P* < 0.05). HE staining was then used for *cortical damage* observation (Figure 3b). The cortex structure of sham group was normal, no edema, dense structure, clear cell outline and nucleolus, and no solid atrophy; however, the histopathology of the MCAO group was abnormal, most of the neurons were necrotic, disordered, and large vacuoles were produced. On the contrary, pre-ischemic exercise ameliorated these pathological abnormalities. These results demonstrated that pre-ischemic exercise alleviated *brain injury*, as well as reduced the score of neurological function injury in MCAO rats.

### 3.3 Pre-ischemic exercise inhibits the activation of JNK and ERK1/2 in MCAO rats

**Figure 4:**
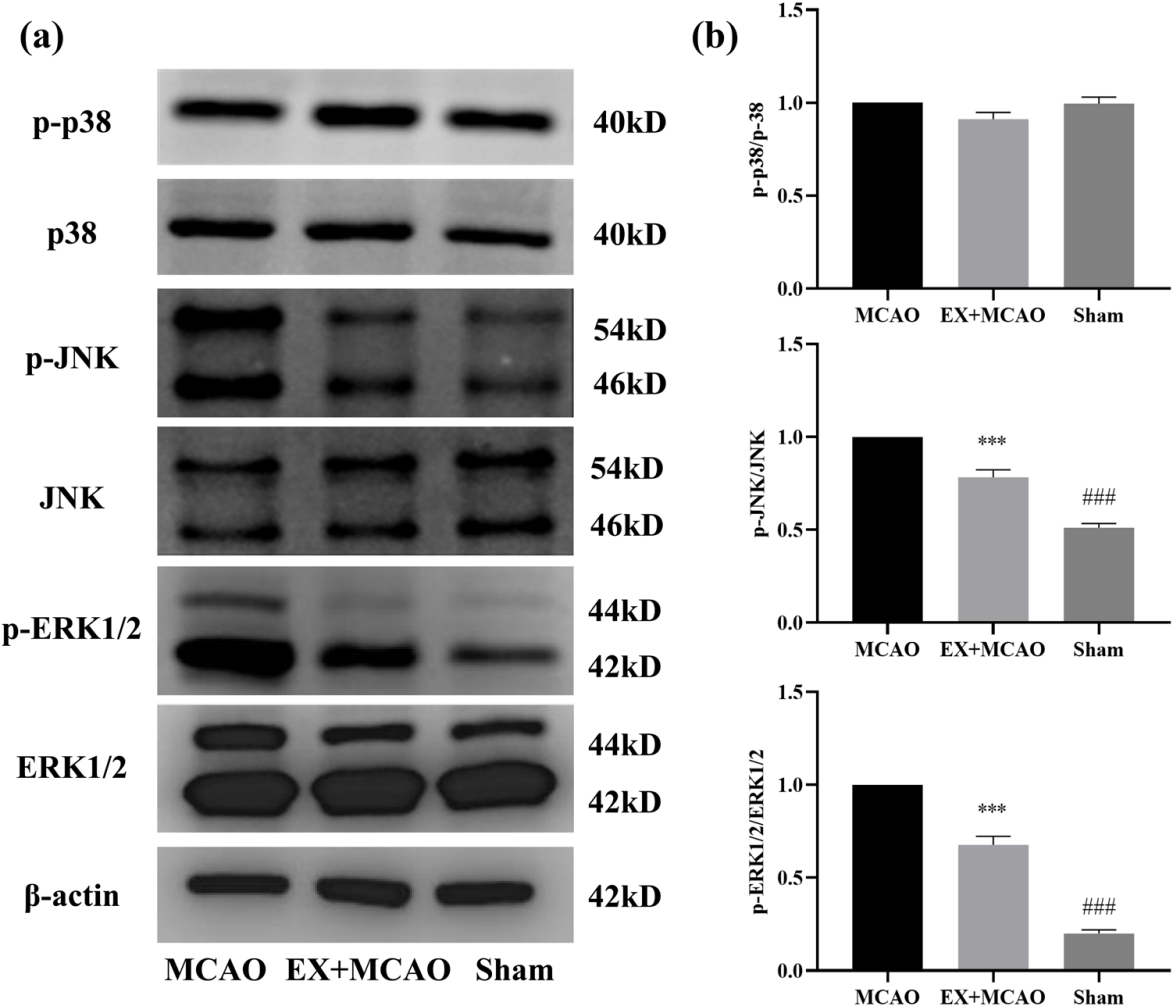
Effects of pre-ischemic exercise on MAPK pathway in MCAO rats. (a) Representative western blot for p-p38, p-38, p-JNK, JNK, p-ERK1/2, and ERK1/2 in the infarcted cortex of three groups. (b) Quantification of p-p38/p-38, p-JNK/JNK, and p-ERK1/2/ERK1/2 protein levels, normalized to β-actin. *n* = 6/group, **P < 0.01 vs MCAO group, ***P < 0.001 vs MCAO group, ^##^ *P* < 0.01 vs EX + MCAO group, ^###^ *P* < 0.001 vs EX + MCAO group. Data were expressed as mean ± SD.

Subsequently, we attempted to verify the effects of pre-ischemic exercise on MAPK pathway expressions under cerebral ischemic conditions. Western blot analysis showed that (Figure 4a and b) MCAO-treated rats exhibited increased expression of phosphorylation of ERK1/2 and JNK; however, phosphorylation of p38 has no difference among all three groups. The EX + MCAO group showed a significant decrease in p-JNK and p-ERK1/2 relative to the MCAO group. Those histological findings suggest that EP inhibited the activation of I/R-induced MAPK signaling pathway.

### 3.4 Pre-ischemic exercise suppresses the levels of microglia and proinflammatory cytokines in serum

**Figure 5:**
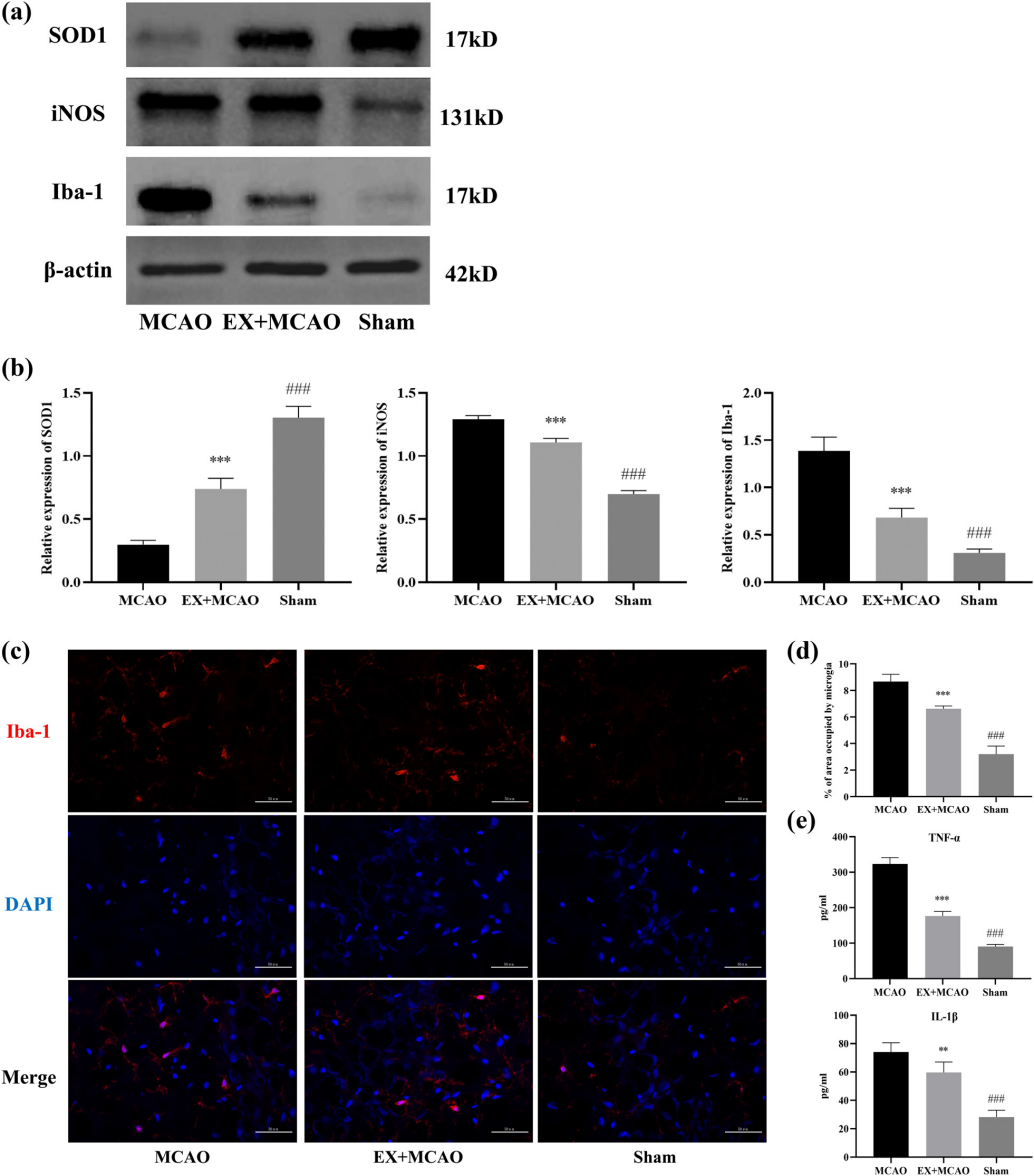
Effects of pre-ischemic exercise on neuroinflammation in MCAO rats. (a) Representative western blot for SOD1, iNOS, and Iba-1 in the infarcted cortex of three groups. (b) Quantification of SOD1, iNOS, and Iba-1 protein levels, normalized to β-actin. *n* = 6/group, ***P < 0.001 vs MCAO group, ^###^ *P* < 0.001 vs EX + MCAO group. Data were expressed as mean ± SD. (c) Representative immunofluorescence images shown activated microglia (red) in the infarct cortex of each group. Scale bar = 50 µm. (d) Histograms showing activated microglia in each group. *n* = 6/group, ***P < 0.001 vs MCAO group, ^###^ *P* < 0.001 vs EX + MCAO group. Data were expressed as mean ± SD. (e) ELISA shown the levels of serum TNF-α and IL-1β in rats. *n* = 6/group, **P < 0.01 vs MCAO group, ***P < 0.001 vs MCAO group, ^###^ *P* < 0.001 vs EX + MCAO group. Data were expressed as mean ± SD.

We first analyzed the expression of SOD1, iNOS, and Iba-1 in the infarct cortex by western blot and found that pre-ischemic exercise significantly attenuated the increased levels of SOD1, iNOS, and Iba-1 caused by MCAO surgery (Figure 5a and b). And immunofluorescence results show that activated microglia was increased by MCAO surgery, which could be reversed by pre-ischemic exercise (Figure 5c and d). Activated microglia secrete various neurotoxic cytokines, such as TNF-α and IL-1β. We speculated that pre-ischemic exercise reduces the release of pro-inflammatory cytokines by inhibiting microglia activation. Next, we measured the levels of inflammatory factors in rats brain tissues. Accordingly, the level of TNF-α and IL-1β was increased in comparison to sham rat, while those inflammatory factors in the EX + MCAO group were lower than those in the MCAO group (Figure 5e).

### 3.5 Pre-ischemic exercise inhibits apoptosis in MCAO rats

**Figure 6:**
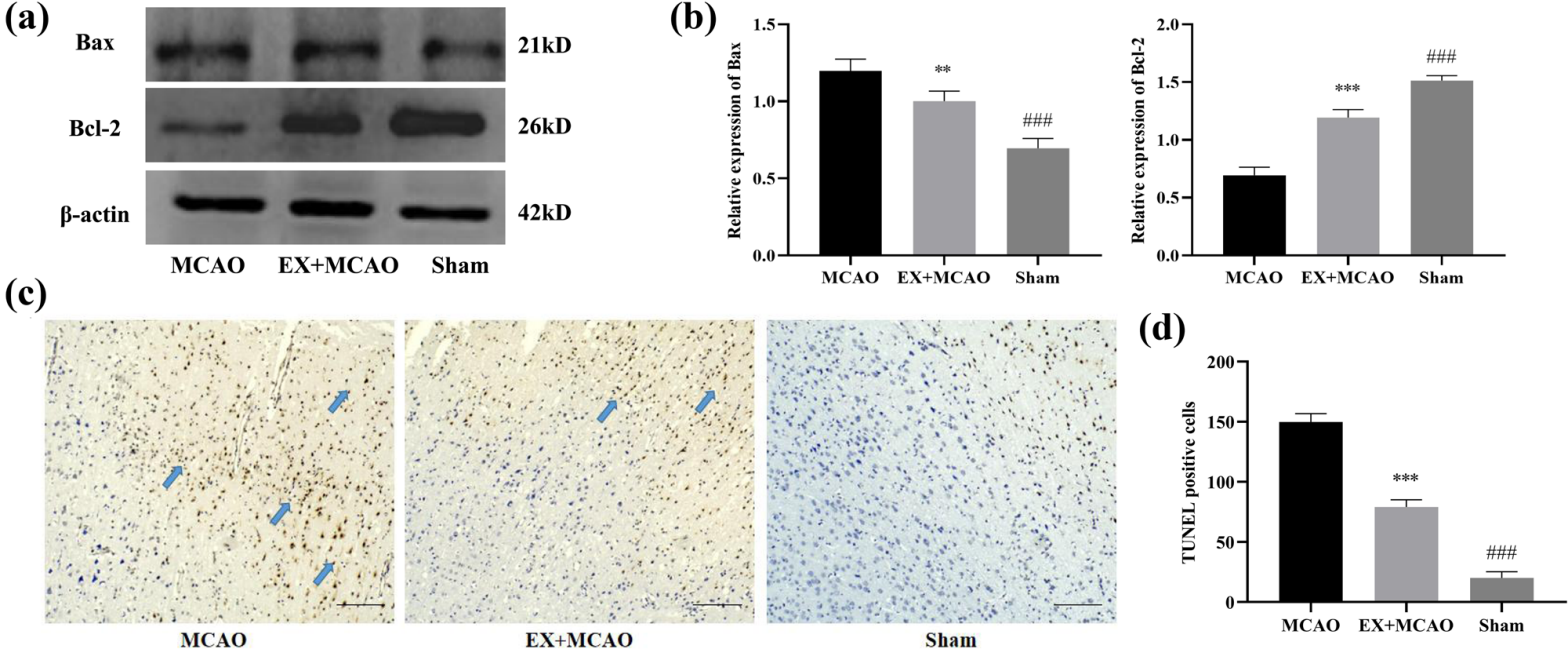
Effects of pre-ischemic exercise on apoptosis in MCAO rats. (a) Representative western blot for Bax and Bcl-2 in the infarcted cortex of three groups. (b) Quantification of Bax and Bcl-2 protein levels, normalized to β-actin. *n* = 6/group, **P < 0.01 vs MCAO group, ***P < 0.001 vs MCAO group, ^###^ *P* < 0.001 vs EX + MCAO group. Data were expressed as mean ± SD. (c) Representative images shown the TUNEL-positive cells in the infarct cortex. Scale bar = 200 µm. (d) Histograms showing the number of infarct cortical TUNEL-positive cells in each group. *n* = 6/group, ***P < 0.001 vs MCAO group, ^###^ *P* < 0.001 vs EX + MCAO group. Data were expressed as mean ± SD.

Next, we sought to test whether the MAPK signaling pathway affects the cell apoptosis of in ischemic stroke rats. At first, we analyzed Bax (proapoptotic protein marker) and Bcl-2 (antiapoptotic protein marker) expression by western blot analysis. Bax expression from the EX + MCAO group led to a decrease compared to the MCAO group (Figure 6a and b), and compared with the MCAO group, the EX + MCAO group significantly increased the protein level of Bcl-2 (Figure 6a and b). In addition, we evaluated the number of apoptotic cells by TUNEL staining (Figure 5c and d). The results showed that the number of TUNEL double-positive cells was elevated in rat models of MCAO but reduced in rat treated with EP in comparison to control rats.

### 3.6 Correlations analysis

**Figure 7:**
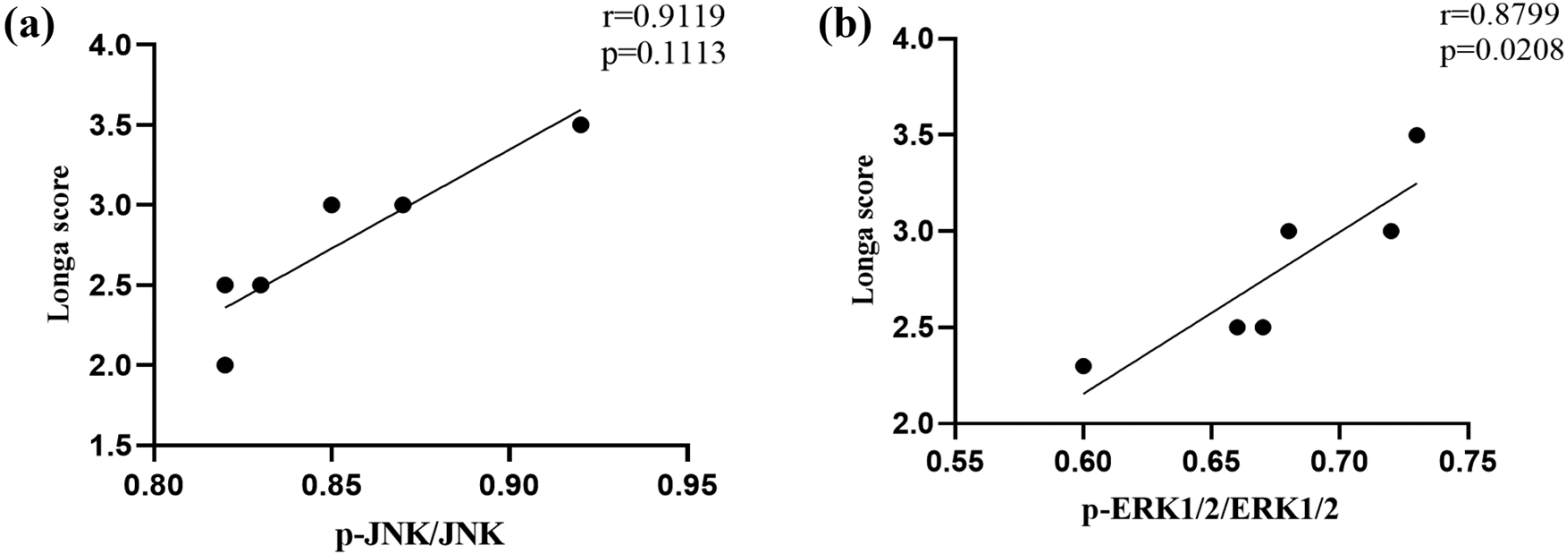
Correlation analysis. (a) Correlation between neurological function and p-JNK/JNK levels on the third day after MCAO operation. (b) Correlation between neurological function and p-ERK1/2/ERK1/2 expression on the third day after MCAO operation. *P* < 0.05 was considered statistically significant.

The Pearson’s test found that Longa score was positively correlated with the p-JNK/JNK expression level at 3 days after MCAO (*r* = 0.9119, *P* = 0.1113, Figure 7a), and the Longa score was positively correlated with the p-ERK1/2/ERK1/2 expression level (*r* = 0.8799, *P* = 0.0208, Figure 7b). The data showed that the activation of MAPK pathway is closely related to the functional recovery in MCAO animals.

## 4 Discussion

The study demonstrated the neuroprotective effects of pre-ischemic exercise in acute ischemic stroke. We found that exercise pre-treatment ameliorated infarct volume, neurological function, and brain injury after cerebral I/R injury in rats. More importantly, exercise significantly suppressed various severe inflammatory responses and dampened the upregulation of apoptosis protein expression. The underlying mechanism was partially related to the inhibition of MAPK pathway.

Physical exercise is an effective way to improve the brain’s tolerance to ischemic injury. The concept of EP was first described by Yamashita et al. in 1999 [14]. Since then, numerous studies have found that EP could effectively reduce brain injury, maintain the integrity of the blood–brain barrier, reduce the excitotoxicity of glutamate, and promote neurogenesis and angiogenesis [13,15,16]. However, few studies investigated the underlying mechanisms by which EP regulate ischemic injury. It has been reported that the activation of MAPK pathway is closely related to ischemic stroke [17]. And compared with voluntary running wheel, forced treadmill exercise can significantly increase CBF and reduce acute brain injury in rats with cerebral ischemia [18]. Therefore, in this study, the change in MAPK pathway in peri-infarct area after cerebral ischemia was taken as the object, the pre-ischemic forced treadmill exercise was used as the intervention way, so as to explore the mechanism after forced treadmill exercise promoting the recovery of rats after I/R injury.

MAPK is an important signaling pathway that regulates cell proliferation, differentiation and apoptosis and migration [19]. In this study, we found that the levels of p-ERK1/2 and p-JNK in the peri-infarct cortex of MCAO rats showed a rapid upward trend, and the ratio of p-ERK1/2/ERK1/2 to p-JNK/JNK was significantly increased (Figure 4). This finding is consistent with other previous studies [20]. The inhibitions of three subgroups (ERK1/2, p38, and JNK) of MAPK pathway could produce a protective effect in stroke. MCAO rats were injected intraperitoneally with U0126, a specific inhibitor of MEK (MAPK/ERK kinase), and sacrificed 24 h later. It was found that U0126 treatment significantly improved brain injury and neurological symptoms and decreased the level of p-ERK1/2 and downstream targets p-Elk-1 [21]. Besides, the role of the MAPK/ERK1/2 pathway in ischemic stroke is controversial. Because ERK1/2 is involved in the repair of exogenous growth factors and estrogens in the ischemic brain, numerous studies attribute a protective role to its activation. There are other studies support that its neuroprotective role is due to its inhibition could promote inflammation and oxidative stress [22]. Previous studies have shown that p-ERK1/2 expression is significantly decreased in early brain injury following pre-stroke exercise training [23]. Therefore, it is reasonable that treadmill exercise significantly inhibited the activation of MAPK pathway 3 days after cerebral ischemic injury in this experiment.

Acute brain damage after ischemia is usually related to the activation of microglia, which are major cell to post-injury inflammation [24], and M1 (classical activation phenotype) microglia will lead to brain damage through producing pro-inflammatory and neurotoxic cytokines, such as TNF-α and IL-1β, their levels peaking at 24 or 48 h after stroke [25,26]. We found that the activation of M1 microglia and the content of TNF-α and IL-1β were reduced in the EX + MCAO group compared to the MCAO group (Figure 5). Previous study has also demonstrated that physical activity preconditioning can reduce the expression of pro-inflammatory factors during I/R [27]. And the downregulation of MAPK signaling is thought to inhibit the activation of microglia, thereby reducing neuroinflammation[28]. We conclude that EP downregulates the expression of MAPK pathway to reduce inflammatory response after acute brain injury.

Neuronal cell death occurs in cortical regions after I/R injury, and neuronal apoptosis is one of the important pathological changes in early brain injury. Bcl-2 blocks cell necrosis and apoptosis though inhibiting free radical formation and the proapoptotic actions of Bax and Bad [29]. Bax is a proapoptotic gene, which increases the permeability of mitochondrion and inhibits anti-apoptotic protein of Bcl-2 [30]. Our western blot results were consistent with previous studies, with a significant decrease in Bax expression and an increase in Bcl-2 expression in the peri-infarct cortex [31]. In addition, our TUNEL staining results revealed a significant decrease in the number of apoptotic cells in the EX + MCAO group (Figure 6). As a mechanism, we focused on the MAPK pathway, which plays an important role in inhibiting apoptosis following ischemic injury. In the present study, we demonstrated that neuronal apoptosis after I/R injury may be related to the expression of MAPK pathway inhibited by EP (Figure 7).

## 5 Conclusion

In summary, pre-ischemic exercise reduces acute brain damage after MCAO. The underlying mechanism could be through suppressing M1 microglia-mediated pro-inflammatory cytokines and cell apoptosis through inhibiting MAPK pathway. On the basis of these results, we suggest that EP might represent a promising therapeutic strategy for acute brain injury after ischemic stroke.

## Abbreviations

MAPK: mitogen-activated protein kinase
ERK1/2: extracellular signal-regulated kinase 1/2
JNK: jun amino-terminal kinase
MCAO: middle cerebral artery occlusion
EX: exercise
CBF: cerebral blood flow
HE: hematoxylin and eosin staining
I/R: ischemia–reperfusion
EP: exercise preconditioning
BBB: blood–brain barrier
iNOS: inducible nitric oxide synthase
SOD1: superoxide dismutase 1
